# Detecting sequence polymorphisms associated with meiotic recombination hotspots in the human genome

**DOI:** 10.1186/gb-2010-11-10-r103

**Published:** 2010-10-20

**Authors:** Jie Zheng, Pavel P Khil, R Daniel Camerini-Otero, Teresa M Przytycka

**Affiliations:** 1 Computational Biology Branch, NCBI, NLM, National Institutes of Health, 8600 Rockville Pike, Bethesda, MD 20894, USA; 2 Genetics and Biochemistry Branch, NIDDK, National Institutes of Health, 5 Memorial Drive, Bethesda, Maryland 20892, USA

## Abstract

**Background:**

Meiotic recombination events tend to cluster into narrow spans of a few kilobases long, called recombination hotspots. Such hotspots are not conserved between human and chimpanzee and vary between different human ethnic groups. At the same time, recombination hotspots are heritable. Previous studies showed instances where differences in recombination rate could be associated with sequence polymorphisms.

**Results:**

In this work we developed a novel computational approach, LDsplit, to perform a large-scale association study of recombination hotspots with genetic polymorphisms. LDsplit was able to correctly predict the association between the FG11 SNP and the DNA2 hotspot observed by sperm typing. Extensive simulation demonstrated the accuracy of LDsplit under various conditions. Applying LDsplit to human chromosome 6, we found that for a significant fraction of hotspots, there is an association between variations in intensity of historical recombination and sequence polymorphisms. From flanking regions of the SNPs output by LDsplit we identified a conserved 11-mer motif GGNGGNAGGGG, whose complement partially matches 13-mer CCNCCNTNNCCNC, a critical motif for the regulation of recombination hotspots.

**Conclusions:**

Our result suggests that computational approaches based on historical recombination events are likely to be more powerful than previously anticipated. The putative associations we identified may be a promising step toward uncovering the mechanisms of recombination hotspots.

## Background

Meiotic recombination is an important cellular process. Errors in meiotic recombination can result in chromosomal abnormalities that underlie diseases and aneuploidy [1,2]. A main driving force of evolution, recombination provides natural new combinations of genetic variations. Recombination events tend to cluster into narrow spans of a few kilobases long, called 'recombination hotspots', which have been observed in the human genome [3,4] as well as in other species [5-7]. Understanding recombination hotspots can provide insight into linkage disequilibrium patterns and help create an accurate linkage map for disease-association studies. Despite the importance of meiotic recombination hotspots, the mechanism behind them is still poorly understood. Intriguing questions remain to be answered: for example, how the hotspots are originated, how their locations and intensities are regulated, how inheritable they are, and so on.

There are three methods for estimating recombination rates. Sperm-typing is an experimental method that allows the recombination rate for an individual man to be measured [8]. It has highly sensitivity due to a large number of sperm cells analyzed. However, it can only be used for short genomic regions due to limitations on the PCR product size and multiplexing. The second method to identify recombination events uses pedigree data [9-11]. This method allows genome-wide recombination rates to be studied, and allows identification of recombination events in individuals. At present, however, the pedigree-based method has a low resolution and a high variance due to the usually low number of meioses examined. Since recombination hotspots are usually a few kilobases wide, it is difficult to accurately detect hotspots with the current techniques of pedigree studies. The third method is the inference of historical recombination rates by studying linkage disequilibrium (LD) patterns using a coalescent model [4,12]. As high-throughput, genome-wide and dense SNP data are available from the HapMap project [13,14], the LD-based method is gaining more popularity. This approach allows for high resolution genome-wide studies. It is cheap, relatively fast, and provides clues about evolutionary history. An important caveat related to this method is that the computed rates are averaged over thousands of past generations. However, since the majority of hotspots persist over thousands of generations and there is a good agreement between the experimental and 'historical' hotspots, computationally derived hotspots provide a good representation of hotspots in the population [12,15].

Using the above methods, extensive variation in recombination hotspots has been observed across species, implying that hotspots evolve rapidly [16,17]. Despite over 98% sequence identity between the human and chimpanzee genomes, there is no correlation in the positions of their hotspots [18-20]. Differences in recombination also exist among different human ethnic groups [3,21,22]. Moreover, there is evidence for inter-individual variation in recombination [10,23].

This interplay between conservation and variability has been difficult to model. One model explaining the rapid evolution of recombination hotspots is the biased transmission of non-hotspot alleles, as a result of which a hotspot tends to disappear [24,25]. This model, however, is in conflict with the fact that recombination hotspots persist for many generations, which leads to the 'hotspot paradox' [26,27]. Various models have been proposed to solve the paradox [27-29]. In particular, it has been proposed that the hotspot paradox can be explained by a combination of *cis*- and *trans*-acting elements that jointly influence hotspot activity [29,30].

One approach to correlating recombination with sequence features is to divide the genome into regions of high recombination rates (called 'jungles') and low recombination rates (called 'deserts'), and then measure the correlation by comparing the enrichment for candidate elements in jungles and deserts. Using this method and LD-based historical recombination hotspots in human, Myers *et al. *[12] observed some motifs that are enriched in hotspots, among which CCTCCCT and CCCCACCCC are the most prominent. Applying a similar method to mouse data, Shifman *et al. *[31] observed an enrichment for the same two motifs as well as repeats. More recently, using the phase 2 HapMap data, Myers *et al. *[32] extended the CCTCCCT motif to a family of motifs based around the degenerate 13-mer CCNCCNTNNCCNC, which was found to occur in about 40% of human hotspots. Examining the variation of recombination rates across either the genome or populations, studies have shown a correlation between recombination and genomic regions of special properties (for example, GC content, chromatin structure) [12,14,33]. None of these elements, however, can consistently explain the presence of recombination hotspots.

Pedigree-based methods have been used to search for sequence polymorphisms associated with genome-wide recombination phenotype. Kong *et al. *[11] identified three SNPs that are associated with high recombination rate in males, but associated with low recombination rate in females. Interestingly, the three SNPs are located in the *RNF212 *gene, a putative ortholog of the *ZHP-3 *gene in *Caenorhabditis elegans *whose functions are involved in recombination and chiasma formation. Chowdhury *et al. *[34] identified six genetic loci associated with recombination phenotype, including one in the *RNF212 *gene, and also found differences in sequence polymorphisms associated with male and female recombination.

Molecular experimental approaches have also been used to predict *trans*- and *cis*-factors of recombination hotspots. Using a PCR-based method on mouse germ-lines, Baudat and de Massy [30] identified a *trans*-acting element that activates by 2,000-fold the recombination activity of a hotspot near the *Psmb9 *gene in the mouse major histocompatibility complex, as well as a *cis*-acting element that represses the hotspot. By comparing crossover rates in very short regions among different males using sperm genotyping experiments, Jeffreys and Neumann [24,35] identified SNPs inside two hotspots (DNA2 and NID1) such that individuals with a particular genotype at such a SNP have a much higher recombination rate at the corresponding hotspot than other individuals; that is, the alleles of such a SNP correlate with the variation of recombination rate. Interestingly, one of these SNPs is located within CCTCCCT, one of the aforementioned motifs [12]. It is known that the mouse *Prdm9 *gene is uniquely expressed in early meiosis, capable of trimethylation of histone H3 lysine 4, and has a role in infertility and double-strand break repair [36]. Recently, three groups of researchers identified Prdm9 as a *trans*-acting protein for recombination hotspots of human and mouse [37-39]. Importantly, human Prdm9 protein was predicted to recognize the aforementioned 13-mer motif CCNCCNTNNCCNC in a zinc finger binding array. The fast evolution of Prdm9 protein and its binding motif can explain the lack of hotspot conservation between human and chimpanzee [39]. Even more recent work of Berg *et al. *[40] demonstrated that human sequence variation in the *Prdm9 *locus has a strong effect on sperm hotspot activity. However, since the 13-mer motif occurs in only about 40% of human hotspots [32] and the variation in the zinc finger array of the *Prdm9 *gene can explain only about 18% of variation in human recombination phenotype [38], it is unlikely that the 13-mer motif and the Prdm9 protein are the sole regulators of recombination hotspots.

In this work we investigated whether SNP population data, such as that in the HapMap database, could be used to uncover associations between differences in hotspot strength and sequence polymorphisms. Hellenthal *et al. *[41] argued that such genotype-dependent recombination may be difficult to uncover due to biased gene conversion (BGC). Specifically, they argued that it cannot be guaranteed that a chromosome that is cold in the current generation underwent a smaller number of recombinations in the past than a chromosome that is currently hot. The argument of Hellenthal *et al. *as well as other comparisons between LD patterns and sperm typing observations [42] highlights the difficulty of the problem, but it does not exclude the possibility that meaningful associations can be identified.

We developed a simple method called LDsplit that divides the population of chromosomes into two subpopulations by SNP alleles (that is, all members in each set have the same allele at that SNP), estimates the recombination rates for both subpopulations of chromosomes, and compares the difference between these rates to the difference expected by chance. To correct for potential bias due to different allelic backgrounds, we standardized the hotspot difference of each hotspot-SNP pair by the empirical distribution of SNPs with the same minor allele frequency (MAF) in a chromosome.

First, running on HapMap SNP data, LDsplit was able to uncover the known association between the FG11 SNP and the DNA2 hotspot [24], with the strongest association in the larger set of combined Chinese and Japanese populations (CHB + JPT). Then, we used simulation to show that LDsplit was robust to confounding evolutionary factors of recurrent mutation and BGC. Running LDsplit on the SNP data of human chromosome 6 of Chinese and Japanese populations (CHB + JPT), HapMap phase II, we found that 15.36% (120 out of 781) tested recombination hotspots are associated with at least one SNP. We showed that this is unlikely to occur by chance, and unlikely to be due to LD patterns generated by different allelic backgrounds or selective sweep. We extended the identified SNPs to flanking regions and found enriched elements, such as self-chains and open chromatins. In addition, we identified an enriched motif, GGNGGNAGGGG, whose complementary sequence partially matches the 13-mer motif CCNCCNTNNCCNC, which was previously reported to be critical in recombination hotspots [32,37].

Our results suggested that LD-based computational methods for associating sequence polymorphisms with recombination hotspots are likely to be more powerful than previously anticipated. Moreover, the putative associations that we identified using LDsplit would be an important step toward uncovering regulatory mechanisms of recombination hotspots. The hotspot-SNP pairs in chromosome 6 of the HapMap CHB + JPT population and their LDsplit *q*-values are available in Additional file 1. The computer source code of LDsplit and simulation is freely available in Additional file 2, or can be downloaded from the LDsplit website [43].

## Results

### Outline of LDsplit

We first provide an overview of the LDsplit approach. Technical details of the approach are provided in the Materials and methods section. For each candidate SNP, LDsplit divides the population of chromosomes into two subpopulations: one subpopulation containing chromosomes having allele 0 of this SNP, and the other subpopulation having allele 1. If the SNP is associated with the hotspot, then different alleles of the SNP may putatively correspond to different levels of recombination activities in the hotspot. For example, while one allele could enhance the hotspot, the other allele could suppress it. Using the LDhat method we estimated the population recombination rate *ρ *= 4*N*_*e*_*r *for each segment (that is, the region between two consecutive SNPs), and the recombination activity of a segment is measured by the product of *ρ *and physical length of the segment. The recombination activity of a hotspot, also called hotspot 'strength', was then measured by the sum of recombination activities of the segments that the hotspot spans. Since the actual level of hotspot strength in each chromosome is unknown, we used the difference of historical hotspot activities between the two subpopulations as a proxy for the current hotspot differences between the subpopulations (see Materials and methods for details). Let *ρ*_0 _and *ρ*_1 _denote the strengths of the same hotspot of two different subpopulations, then the difference of recombination activities between the two subpopulations, denoted Δ*ρ*, is defined as (*ρ*_0 _- *ρ*_1_)/(*ρ*_0 _+ *ρ*_1_), that is, the difference of hotspot strengths normalized by the sum. To measure the significance of a hotspot-SNP association, we estimated the *P*-value of the alternative hypothesis that the observed Δ*ρ *is non-zero, using permutation tests (see Materials and methods). In computing *P*-value, we assumed that the Δ*ρ *from the random split should be normally distributed around zero. We used the Shapiro test to filter out the hotspots that violated this assumption. However, we observed that hotpots with non-normal distributions of random Δ*ρ *typically contain a few 'outlier' chromosomes. We developed a method to identify such outlier chromosomes (see Materials and methods section for details) and observed that after their removal from the population, the distribution of Δ*ρ *often passed the normality test.

There might be a potential bias in estimating differences in recombination rates as a result of the frequency difference between the two alleles of a SNP. The allele with lower frequency tends to be younger and its subpopulation is likely to have stronger LD around the SNP than the allele with higher frequency [44]. Moreover, the younger allele has less time to accumulate historical crossover events, which makes it harder for LDhat to detect a hotspot in that sample. As a result, the more frequent allele of a candidate SNP tends to appear 'hotter' than the rare allele. This trend has been indeed observed in our data set (not shown). To control for such artifacts, we adopted a strategy similar to [44] as follows. First, let us define Δ*ρ *as the *ρ *of the more frequent allele minus the *ρ *of the rare allele. Then, for each hotspot-SNP pair, we estimated the expectation, denoted *E*(Δ*ρ*), and standard deviation of Δ*ρ*, denoted SD(Δ*ρ*), from the empirical distribution of those SNPs with equal MAF values from the chromosome that contains the hotspot-SNP pair. Then, the standardized version of hotspot difference is defined as (Δ*ρ *- *E*(Δ*ρ*))/SD(Δ*ρ*). We applied the same standardization to the permutation data, and obtained the standardized *P*-values.

### Sperm typing case study

We first tested if LDsplit was able to correctly predict a hotspot-SNP association that had been shown to exist by sperm typing experiments [24], namely the FG11 SNP with the DNA2 hotspot in the MHC class II region. It was observed that individuals with the TT or TC allele at the FG11 SNP have a recombination rate about 20 times higher than those with the CC allele. Hence, we call the T allele 'hot' and the C allele 'cold'. Interestingly, FG11 is located in the aforementioned CCTCCCT motif [12]. Moreover, it was reported that recombinant meioses from heterozygous individuals were more likely to have the T allele (68 to 87%) than the C allele, indicating the existence of BGC at the DNA2 hotspot. Hellenthal *et al. *[41] used the DNA2 hotspot and the FG11 SNP as an example to argue that, due to BGC, it might be difficult to uncover such differences in recombination rates between hot and cold alleles using an LD-based method.

**Figure 1:**
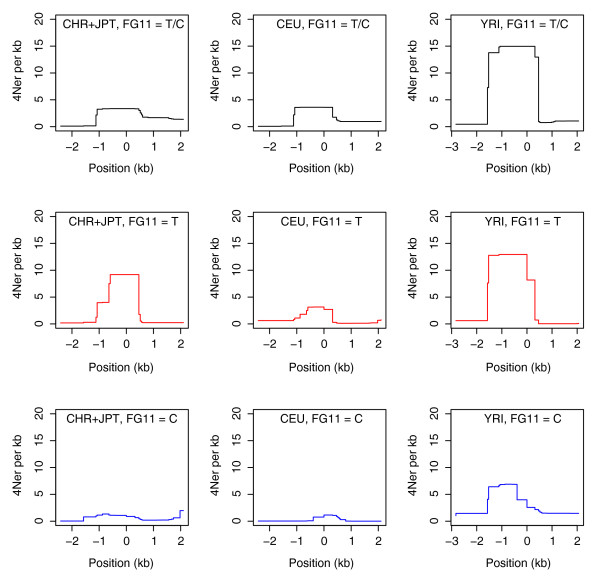
**Profiles of recombination rate at the DNA2 hotspot in the MHC region in chromosome 6 of the three populations (HapMap phase II)**. For simplicity, we set the position of FG11 SNP at 0. The DNA2 hotspot spans from about -1 kb to 0.5 kb. In each population, the top profile is from the whole sample (T or C allele at FG11); the middle profile is from the subpopulation with the T allele (hot); the bottom profile is from the subpopulation with the C allele (cold). The population and the alleles at FG11 are labeled above each plot.

Despite the presence of BGC, however, LDsplit was able to confirm the sperm typing result. As shown in Figure 1, the 'hot' T allele indeed has a higher population recombination activity at the DNA2 hotspot (estimated by LDhat) than the 'cold' C allele. The small recombination rate of the C allele is unlikely to be due to the artifact of a small sample size because in the CHB + JPT (Han Chinese in Beijing, China and Japanese in Tokyo, Japan) population there are more chromosomes with the C allele than with the T allele (117 versus 63), and in the other populations the numbers of chromosomes with C versus T alleles are similar (58 versus 62 in CEU (Utah residents with Northern and Western European ancestry) and 51 versus 69 in YRI (Yoruba in Ibadan, Nigeria)). Moreover, as shown in the last column of Table 1, the association between the SNP FG11 and the hotspot DNA2 is statistically significant in the CHB + JPT (*P *< 0.000447) and the YRI (*P *< 0.0235) populations. In the CEU population, the association is not statistically significant, but the T allele still has a higher population recombination rate than the C allele, consistent with those in the other populations (Figure 1). We noticed that in this case the distribution of Δ*ρ *in random permutations was not normal (see *P*-values of Shapiro's tests in Table 1; note that a small *P*-value for the normality test indicates that the distribution deviates from the normal distribution). Therefore, we identified the outlier chromosomes and removed them from the corresponding populations. After the removal of the outlier chromosomes, we observed: (1) the distribution of Δ*ρ *passed the normality test; (2) the association between FG11 and DNA2 in the CHB + JPT population became even more significant, and the association in the YRI population also became significant (Table 1). We repeated multiple runs for each population and obtained consistent results (data not shown). The case study result implies that, despite complicating factors such as BGC, it is possible, at least in some cases, to use a computational approach based on historical recombination rates to identify the associations of sequence polymorphisms with allele-specific recombination hotspots.

**Table 1:** Effect of removing outliers in the case study of the DNA2 hotspot and FG11 SNP

			Before removal of outliers		After removal of outliers	
				
Population	Outlier chromosome	Grubbs *P*-value for outlier	Shapiro *P*-value (normality of Δ*ρ*)	Association *P*-value for FG11	Shapiro *P*-value (normality of Δ*ρ*)	Association *P*-value for FG11
CHB + JPT	29	6.6e-7	0.00193	0.006258	0.5014	0.0004474
	32	6.9e-6				
	56	0.051				
CEU	102	0.00111	0.003336	0.08024	0.3915	0.2129
YRI	116	0.03	0.04887	0.1884	0.1302	0.02349
	52	0.028				

In addition, we tested LDsplit on another sperm typing case. It was reported that sperm typing analysis could not find any local polymorphisms associated with the variation in crossover rate in hotspots MSTM1a and MSTM1b on human chromosome 1 [45]. Since the two hotspots are within 2 kb of each other, and HapMap SNPs at this region are not dense enough to distinguish them, we consider them as one hotspot. We applied LDsplit on the 200-kb region around the hotspot, and found no SNPs with a *P*-value <0.01 within the 200-kb window. The nearest SNPs with *P*-values <0.05 for the CEU, CHB + JPT and YRI populations are about 7 kb, 13 kb and 8 kb away from the hotspot. This result is consistent with the lack of local associated polymorphisms observed by sperm typing. It might be possible that there are associated SNPs among the SNPs with *P*-values <0.05. However, due to the relatively low resolution of HapMap SNPs near this hotspot compared with the sperm typing data, the putative association suggested by LDsplit may not have high confidence.

### Simulation study

The recombination history might be quite complicated and it is possible that a chromosome that is cold in the current generation underwent more crossovers in the past than a currently hot chromosome. To test whether LDsplit is able to detect signals of hotspot-SNP association from the LD patterns, we carried out forward simulations of crossover and BGC in which the causal SNP and its hot and cold alleles were specified (see Materials and methods section for details). Running on simulated SNP data, LDsplit calculated for SNPs with MAF ≥ 0.3 (including the causal SNP) the *P*-values indicating the strength of association with the simulated hotspot. When the hot allele frequency of causal SNP in the population was close to 0.3, it could happen that its MAF in a sample was lower than 0.3. Such rare cases would be discarded from evaluation.

We tested different values of key parameters, namely the positions of causal SNPs and hot allele frequencies at the beginning and the end of the simulation (Tables S1, S2, and S3 in Additional file 3). If the hot allele frequency at the beginning of evolution was 100%, it is called the 'cooling' model; otherwise, if the beginning hot allele frequency was 0%, it is called the 'heating' model. Both cooling and heating models were simulated. For all the combinations of parameters, we simulated 30 populations, and from each population we randomly sampled 10 subsets, each consisting of 90 individuals (180 haplotypes) as benchmark data. The relatively small numbers of samples per population were due to the high computational cost of LDsplit.

We then evaluated the performance of LDsplit as follows. First, we measured how likely LDsplit was to predict the hot and cold alleles of the causal SNP. If the hotspot strength in the subpopulation of the hot allele was bigger than that of the cold allele, we counted it as a correct prediction of direction. We report the proportion of correct predictions in the samples of a population as a measure of performance. Second, we tested if the LDsplit *P*-value could accurately measure the hotspot-SNP association. If the *P*-value is < 0.05, it is a positive result; otherwise, it is a negative result. The causal SNP is a 'true' result, and all other SNPs are 'false'. To correct for redundancy of SNPs in strong LD, we clustered SNPs into LD blocks (*r*^2 ^≥ 0.8) using the ldSelect program [46], and from each block picked tag SNPs as causal SNPs or otherwise SNPs with the smallest *P*-values. By these criteria, we counted true positive (TP) SNPs as the number of tag SNPs that are both true and positive, and similarly for false positive (FP), true negative (TN) and false negative (FN) SNPs. The sensitivity, specificity, and positive predictive value (PPV) are TP/(TP + FN), TN/(TN + FP) and TP/(TP + FP), respectively. Note that we inserted only one causal SNP while there were usually much more non-causal SNPs, which might amplify the effect of false positives in the calculation of the PPV. For each population, we assessed the above measures of performance among haplotype samples. The average performance of LDsplit on these populations is shown in Table 2. In most cases LDsplit was able to correctly predict the direction of hot versus cold alleles. The sensitivity and specificity are about 60%.

**Table 2:** Average performance of LDsplit on simulation data

Condition	Correct prediction of hot/cold alleles (%)	Sensitivity (%)	Specificity (%)	Positive predictive value (%)
Normal	89.26 ± 18.23	63.15 ± 26.42	58.71 ± 26.53	46.29 ± 22.22
Recurrent mutation	93 ± 9.88	70 ± 27.16	51.78 ± 21.99	43.58 ± 22.49
Long BGC tract (10 kb)	84.29 ± 22.77	53.4 ± 28.34	75.65 ± 12.94	52.60 ± 25.27

The standard deviations are slightly high because we sampled only ten sets of haplotypes for each parameter configuration due to the high computational cost of LDsplit.

In the above simulation, we assumed that the causal SNP was produced by a single mutation event that split the coalescent tree into two subtrees. We consider these simulations to be run under 'normal' conditions. In addition, we tested the robustness of LDsplit under some unusual conditions. The first case is recurrent mutation at the causal SNP. During evolution, multiple mutation events were allowed to occur at the causal SNP after its birth, and its mutation rate was specified to be ten times higher than the background rate. As shown in Table 2, under recurrent mutation at the causal SNP, the accuracy of direction prediction and sensitivity even increases slightly, but specificity and PPV decrease. This result implies that the performance of LDsplit is robust to recurrent mutation. Under the normal conditions, the probability of BGC conditional on a crossover was set to be 50%. As a result, the proportion of recombinant gamete chromosomes with a cold allele from a heterozygous parent would be 75%. Thus, the normal conditions already take into account a quite strong effect of BGC. We next tested LDsplit under more severe BGC by increasing the average length of BGC tract length from 500 bases to 10 kb. As shown in Table 2, LDsplit is robust to more severe BGC effect, and its specificity and PPV even increase, although the sensitivity decreases.

### Large scale analysis

Encouraged by the results for the sperm typing case study and the simulation, we performed a large-scale analysis. First, we identified a list of recombination hotspots from the SNP data for chromosome 6 of the CHB + JPT population of the HapMap dataset, phase II, from which we filtered out hotspots of weak intensity compared to the background (as described in the Materials and methods section). In this way we identified 5,149 hotspots. As mentioned in the outline of LDsplit, to estimate the *P*-values of associations, we assumed that the distribution of random Δ*ρ *(that is Δ*ρ *of random splits into two subpopulations) could be reasonably approximated by the normal distribution. For each hotspot, we estimated the distribution of Δ*ρ *based on 200 random splits. We rejected hotspots with non-normal distributions of random Δ*ρ *(Shapiro's normality test *P *< 0.05), and were left with 781 hotspots.

For each selected hotspot, we considered all SNPs that were within a distance of 200 SNPs on either side of the hotspot and with an MAF of at least 0.3. The lower bound of the MAF value was needed for an accurate estimation of the recombination rate for each subpopulation.

In this study, as in most genome-wide studies where the number of features tested is typically more than tens of thousands, an important concern is multiple testing. To achieve a balance between the number of false positives and the number of true positives, we used the false discovery rate (FDR). The FDR is defined as the expected proportion of false positives among those features claimed to be significant [47]. In addition, to attach a measure of significance to each individual hotspot-SNP association, we mapped every *P*-value to a *q*-value [48]. Specifically, in the set of hotspot-SNP pairs selected by requiring their *q*-values to be no more than α, the expected proportion of false positives (FDR) is also no more than α.

To test further if these hotspot-SNP pairs could have been selected by chance, we simulated the null model (that is, there is no association between hotspots and SNPs) as follows. For each hotspot-SNP pair tested in the real case, we randomly divided the population into two subpopulations whose sizes were equal to the sizes of the real case. Then we calculated *P*-values and *q*-values for these artificial hotspot-SNP pairs, in one-to-one correspondence with the real pairs. As shown in the histograms of real and random *P*-values (Figure S1 in Additional file 3), the vast majority of random *P*-values are uniformly distributed, indicating that they correspond to the truly null hypothesis. Compared with the real case, the set of artificial hotspot-SNP pairs contains fewer small *q*-values and a large number of *q*-values close to 1 (Figure S2 in Additional file 3). This provided additional support that the identification of hotspot-SNP pairs (*q *< 0.01) was not by chance. As shown in Table 3, we observed that 15.36% (120 out of 781) of recombination hotspots were associated with at least one SNP.

**Table 3:** The numbers of hotspot-SNP pairs, and the numbers of hotspots and SNPs involved in those pairs

	Number of hotspot-SNP pairs	Number of hotspots in the pairs	Number of SNPs in the pairs
SNPs outside hotspots			
Total	99,899	781	44,713
Real (*q *< 0.01)	1,430	115	1,361
Random (*q *< 0.01)	85	18	85
Intersection of real and random	45	11	45
SNPs inside hotspots			
Total	1,440	615	1,436
Real (*q *< 0.01)	67	44	67
Random (*q *< 0.01)	4	2	4
Intersection of real and random	3	2	3
SNPs inside or outside hotspots			
Total	101,339	781	44,896
Real (*q *< 0.01)	1,497	120	1,426
Random (*q *< 0.01)	89	18	89
Intersection of real and random	48	11	48

If a hotspot or a SNP is involved in multiple pairs, we counted it only once.

**Figure 2:**
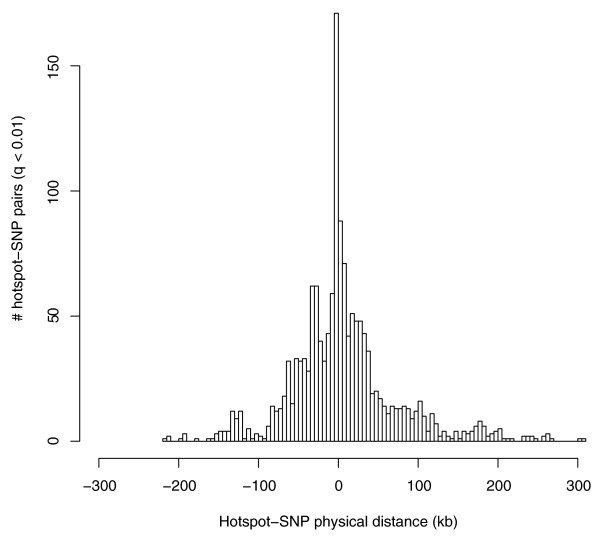
**Distribution of physical distances of candidate hotspot-SNP pairs (*q *< 0.01)**. When a SNP is inside a hotspot, the distance is 0; when a SNP is to the left of a hotspot, the distance is negative.

Next, we studied the distribution of the hotspot-SNP distances of significant hotspot-SNP pairs (*q *< 0.01) measured by: (1) the physical distance (in kilobases) from the SNP to the center of the hotspot; and (2) the number of SNPs between the candidate SNP and the proximal boundary (also a SNP) of the hotspot. Figure 2 shows the distribution of the physical distances. The distances measured by numbers of SNPs show a similar trend (Figure S3 in Additional file 3). LDsplit uncovered more associated SNPs at short distances from the hotspots. We cannot assert to what extent this property should be attributed to the loss of the power of the method over larger distances versus the distribution of the distance from a candidate SNP to an associated hotspot.

As mentioned above, the difference between the recombination rates of the two alleles of a SNP, which is used by LDsplit to assess the significance of association, might be due to different allelic backgrounds; that is, the ancestral allele might have a higher historical recombination rate because it has a longer time to accumulate crossover events than the derived allele. Note that this issue has been addressed, at least in part, by the aforementioned standardization with allele frequencies. In the following, we show that while some effects of the artifact might still exist, they do not dominate the results of LDsplit.

To assess a possible impact of allelic ages on the estimation of recombination rates, we counted the numbers of hotspot-SNP pairs in which the SNP derived allele is 'cold' and the number of such pairs when the derived allele is 'hot'. An allele is called 'cold' when the chromosome sample with that allele has a smaller hotspot strength, and 'hot' otherwise. For simplicity, when a derived SNP allele is cold (or hot), we call the hotspot-SNP pair 'derived-cold' (or 'derived-hot'). The ancestral states of HapMap SNPs were obtained from dbSNP and alignment between human and chimpanzee genomes [44]. Suppose that, despite the standardization with allele frequencies, this artifact still dominates the LDsplit results, then the hotspot-SNP pairs with small *q*-values would be expected to be more enriched with derived-cold pairs than pairs with big *q*-values. However, as shown in Table 4, the pairs with small *q*-values are even less enriched than those with big *q*-values, except when SNPs are outside but within 50 kb of hotspots. Even in the latter exceptional case, the ratio for pairs with *q *< 0.01 is not much bigger than the overall ratio of 1.342. This suggests that the difference in allelic ages did not contribute to small LDsplit *q*-values significantly.

**Table 4:** The numbers of hotspot-SNP pairs in which the SNP-derived allele is cold versus hot

	SNP inside hotspot	**0 <*D ***≤ **50 kb**	**50 kb <*D ***≤ **100 kb**	*D *> 100 kb
*q *< 0.01	34/31 (1.097)	596/354 (1.684)	141/118 (1.195)	92/92 (1.000)
0.01 ≤ *q *< 0.05	55/48 (1.146)	1,066/673 (1.584)	402/271 (1.483)	386/277 (1.394)
0.05 ≤ *q *< 0.5	437/375 (1.165)	11,227/8,030 (1.398)	8,081/6,187 (1.306)	10,182/7,764 (1.311)
*q *≥ 0.5	229/162 (1.414)	6,399/4,877 (1.312)	7,034/5,217 (1.348)	10,164/7,676 (1.324)

Ratios are given in parentheses. The pairs are classified by LDsplit *q*-values and the physical distance *D *between a hotspot and a SNP.

**Figure 3:**
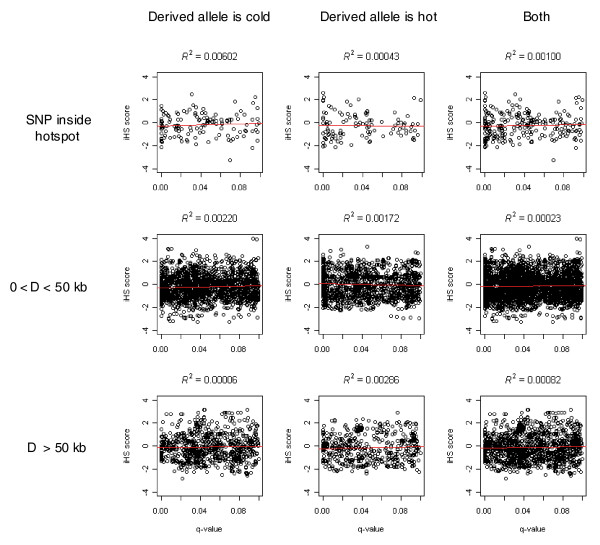
**Scatter plots between LDsplit's *q*-values that are less than 0.1 and Haplotter's iHS scores**. The three columns are, respectively, hotspot-SNP pairs where the SNP-derived allele is cold, hot, and both; the three rows correspond to three ranges of hotspot-SNP physical distances *D*. The red line in each panel is the least square regression line, and *R*^2 ^at the top is the coefficient of determination, measuring the fraction of variance of iHS scores explained by *q*-values.

Some of the hotspot differences might also be caused by the extended haplotype block created by selective sweep at one allele. To estimate the confounding effect between LDsplit and selection, we correlated the LDsplit *q*-values with signals of selective sweep estimated using iHS scores from Haplotter [44]. For a SNP associated with multiple hotspots, we picked the hotspot that is nearest to the SNP. If a large fraction of SNPs identified by LDsplit could be attributed to the signal of selection, there should be a strong positive correlation between the two variables. However, the scatter plots between iHS and *q*-values in Figure 3 suggest that the correlation is weak. The coefficient of determination *R*^2^, which measures the fraction of variance explained, is mostly less than 0.01. The strongest correlation is when SNPs are inside hotspots and the derived allele is cold, with *R*^2 ^= 0.00602. Therefore, most signals of hotspot differences in LDsplit cannot be explained by selective sweep.

### Genomic feature analysis

From the large scale analysis, we identified a list of candidate SNPs associated with recombination hotspots in chromosome 6 of the human genome. In this section, we analyze these SNPs in search of genomic features that might be associated with the regulation of recombination hotspots. After controlling for confounding effects such as hotspot-SNP distance and LD blocks, we selected 498 candidate SNPs and 604 control SNPs (see Materials and methods section for details). The goal was to identify genomic features that preferentially occur near candidate SNPs but not control SNPs.

**Figure 4:**
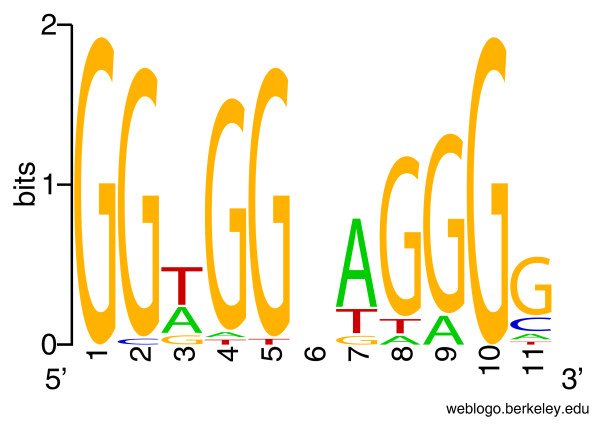
**The 11-mer motif found using MEME to be conserved only around candidate SNPs (sites = 34, E-value = 2.7e-7)**. Its complementary sequence partially matches the CCNCCNTNNCCNC motif previously reported to be associated with recombination hotspots.

First, we searched for conserved motifs near candidate SNPs. The SNPs were extended on both sides to flanking windows of 90 bases long. Running MEME on candidate and control windows, respectively, we identified three motifs in candidate windows and two motifs in control windows. The first two motifs in candidate windows are C-rich and T-rich sequences, and are similar or approximately complementary to the two motifs in the control windows (data not shown). The third 11-mer motif (Figure 4) preferentially occurs around candidate SNPs (sites = 34, E-value = 2.7e-7). Interestingly, its complementary sequence partially matches the well-known 13-mer motif CCNCCNTNNCCNC, which was previously discovered [32] and recently identified as binding sites of the Prdm9 protein [37]. The 90-base windows around candidate SNPs have an average GC% of 0.418 ± 0.0976, slightly higher than the control average GC% of 0.408 ± 0.100 (*P *= 0.0616, Wilcoxon test).

Next, we searched for genomic elements that overlap with windows around candidate SNPs. To catch more complete information, we extended SNPs to windows of 200 bases long. Using the intersection operation of the UCSC genome browser, we counted the proportions of candidate and control windows that overlap with a certain genomic element, and assessed the significance of enrichment by Fisher's test. Of the 20 genomic elements (Table S4 in Additional file 3) we studied, self-chain (alignment of human genome regions with itself indicative of duplications within the genome) and open chromatin (AoSMC DNase Pk) have significant enrichment in candidate windows (Table 5).

**Table 5:** Significant enrichment of genomic elements near candidate SNPs

	Candidate		Control		*P*-value
			
Genomic element	Number of hits	Number of misses	Number of hits	Number of misses	(Fisher's test greater)
Self-chain	178	320	165	439	0.00165
Open chromatin (AoSMC DNase Pk)	25	473	17	587	0.0408
Coding exon	10	488	2	602	0.00789

Overall, there is no difference in enrichment of repeats between candidate and control SNPs in general (Table S6 in Additional file 3). To further analyze particular repeats, we counted the members of the Repeat Masker dataset that overlap with candidate and control windows. The top five repeats that overlap with the highest numbers of candidate windows are not preferentially located near candidate SNPs (Table S6 in Additional file 3). The only repeat with more occurrences near candidate SNPs is MER4D1 (*P *= 0.0414), while (TG)n and MIR3 occur more frequently near control SNPs (*P *= 0.0268).

Ten candidate SNPs fall inside coding exons while only two control SNPs are coding; thus, the majority of candidate and control SNPs are non-coding. There is no significant difference in MAF and ancestral allele frequencies between candidate and control SNPs (data not shown).

Finally, we analyzed the relationship between hotspot-SNP distance and genomic feature enrichment. First, we observed a positive Pearson correlation between hotspot-SNP distances and *q*-values output by LDsplit (*P *= 0.0346; Figure S4 in Additional file 3). The distances have a positive correlation with MAF, and a negative correlation with GC% around candidate SNPs, but neither are significant (Figure S4 in Additional file 3). Furthermore, we compared candidate SNPs within 2 kb of hotspot centers (proximal SNPs) with SNPs 50 kb away (distant SNPs). Similar to the aforementioned analysis using the UCSC genome browser, we counted the numbers of features overlapping with 200-bp windows around proximal and distant SNPs. It turns out that self-chains are more enriched near proximal SNPs than distant SNPs (*P *= 0.00512, Fisher's test), but none of the other elements is significantly enriched or depleted (Table S5 in Additional file 3). However, since only 23 out of 178 SNPs that overlap with self-chains are within 2 kb of hotspots, the enrichment of self-chains reported for all candidate SNPs (Table S4 in Additional file 3) is not due to SNPs within hotspots only. Second, we ran MEME on the 200-bp windows around proximal and distant candidate SNPs but did not find any significantly conserved motif.

## Discussion

Although our approach achieved promising performance on both real and simulation data, it has a few caveats. First, we used historical recombination hotspots inferred from LD patterns to approximate extant hotspots that are needed as phenotypes in such association studies. Thus, we might miss very young hotspots that have no time to leave a signature in the LD patterns, and some hotspots inferred from LD might have already died. However, it has been observed that extant hotspots largely agree with hotspots inferred by LD-based methods [15].

Next, our method looks for single-locus *cis*-association of the variation in hotspot strength with genetic polymorphism in relatively proximal loci. It is possible, as demonstrated in [30], that hotspot activity is influenced by more than one locus including a long range *trans*-effect. The genome-wide study of such epistatic effects and long range *trans*-effects are rather limited due to statistical issues, including multiple testing. In our study this problem is amplified by the computational cost of the permutation test. In the current implementation, each permutation requires re-computing of recombination rates using the computationally intensive LDhat algorithm.

Finally, to accurately estimate the population recombination rate, our computational method requires that both subpopulations are relatively large. Thus, if just a few chromosomes are much hotter or colder than the rest, our method would be less powerful at identifying a corresponding association. For similar reasons, in our large scale study we excluded hotspots for which the distribution of differences in recombination rates in randomly split subpopulations deviated significantly from the normal distribution.

In the sperm typing case study, LDsplit identified the most significant association for the CHB + JPT population, and less significant association for the CEU and YRI populations. This might be due to various reasons. For example, the combined CHB + JPT population has a bigger sample size (90 individuals) than either of the CEU or YRI populations (60 individuals). Moreover, the CEU and the YRI samples have a trio family structure, which may make it more difficult for LDhat to accurately estimate the recombination rates. Another reason might be the difference in demographic history.

## Conclusions

In this work, we demonstrate that the variations in strengths of recombination hotspots could be associated with sequence polymorphisms, and we propose a method called LDsplit to map such associations based on LD patterns in HapMap data. Previous work suggested that it is difficult, if not impossible, to uncover allele-specific recombination hotspots from LD patterns. However, LDsplit was able to correctly predict the association of the FG11 SNP with the DNA2 hotspot in the MHC class II region that had been directly observed by sperm typing experiments. Moreover, we carried out forward simulations of causal SNPs of recombination hotspots, and tested the performance of LDsplit on the simulated data. Despite BGC, the performance of LDsplit turned out to be reasonably good, implying that the extant hot alleles tend to experience more historical crossovers than cold alleles. Then we applied LDsplit to chromosome 6 of the CHB + JPT population and observed widespread associations of sequence polymorphisms with hotspots unlikely to occur by chance. Taking into account the ancestral states of SNPs, we showed that LDsplit is not confounded by the artifact of different allelic backgrounds or selective sweeps. From flanking regions of the SNPs identified by LDsplit with significant association with hotspots, we found a conserved 11-mer motif, whose complement partially matches the 13-mer CCNCCNTNNCCNC, a critical motif for the regulation of recombination hotspots. This result not only confirms previous work [32,37] about the regulatory role of the 13-mer motif, but also demonstrates the utility of LDsplit to find such motifs.

Given the aforementioned restrictions, the LDsplit method is not expected to uncover all associations. Nevertheless, the agreement between the LDsplit prediction and sperm typing result suggests that LDsplit is promising in connecting historical recombination with the extant phenotype of a recombination hotspot. The SNPs predicted by LDsplit may co-segregate with some evolutionarily inheritable factors that regulate the increase and decrease of recombination rates. Therefore, this work should provide an important step towards understanding the regulatory mechanism behind recombination hotspot activity.

## Materials and methods

### Data

We used a subset of the phased data of HapMap phase II, release 22, which consists of 90 JPT and 90 CHB samples of chromosome 6. In total, there are 176,352 SNPs, out of which 56,510 have a MAF ≥ 0.3. We used the latter SNPs for association with hotspots (because SNPs with a MAF that is too small will give small samples of chromosomes for which an LD-based method is not powerful enough to detect recombination hotspots).

Recombination rate profiles were estimated using the program 'interval' from the LDhat package version 2.1 [49]. Hotspots were defined as peaks in the recombination rate profile with widths no more than 20 kb and an average rate above 1 cM/Mb. First, we detected all the peaks in the map (the first derivative is equal to 0 and the second derivative is negative) and fitted a normal distribution to the part of the map from the 50-kb genome region surrounding the center of each peak. Then we extended hotspot boundaries to include all map segments with recombination rates above the mean recombination rate inside the smaller of 2 × fitted peak width, full width at half maximum (FWHM) obtained from the curve fitting, or a 50-kb region centered at the peak. If two adjacent hotspots defined in such a way overlap, we set the peak boundaries in the middle of the valley between the peaks.

### Comparing strengths of a hotspot between two populations

Given a hotspot detected from a combined population (for example, JPT + CHB), we first fixed the boundaries of the hotspot region. Then we estimated the recombination rates of the region for the two subpopulations separately. The two subpopulations could be either true or pseudo. To measure the difference of a hotspot between two subpopulations, we calculated the strength of the hotspot in each subpopulation, denoted by *ρ*. Let *ρ*_0 _and *ρ*_1 _represent the hotspot strengths of the two subpopulations, and define their difference by:

$$\Delta \rho =(\rho_{0}-\rho_{1})/(\rho_{0}+\rho_{1})$$

Then, we standardized the hotspot difference as (Δ*ρ *- *E*(Δ*ρ*))/SD(Δ*ρ*), where the expectation *E*(Δ*ρ*) and standard deviation SD(Δ*ρ*) were estimated from the empirical distribution of SNPs with equal MAF values from the chromosome that contains the SNP in question.

### Permutation tests

To measure the significance of a hotspot-SNP association, we simulated the null hypothesis that there is no difference in hotspot strengths between two split subpopulations, using 200 random permutations. For each permutation, we randomly split the sample of chromosomes into two subsamples each containing at least 30% chromosomes, and then calculated the random Δ*ρ *between the two subsamples as described previously. We estimated the *P*-value of the hotspot-SNP association by the proportion of random |Δ*ρ*| bigger than the observed |Δ*ρ*|.

Due to the formidable computational cost of the permutation tests, we reused the permutation data that one of the authors (PPK) had generated previously - over a month long computation on the NIH Biowulf cluster. In those permutations the chromosomes were divided into two random sets of equal size, and homologous chromosomes of the same person were always permuted together. In the current study, however, we allowed our subpopulations to be of different sizes (albeit still balanced in that the smaller subpopulation consists of at least 30% chromosomes as we considered only SNPs with a MAF of at least 0.3). Furthermore homologous chromosomes of the same individual could be separated. Thus, our permutation test should ideally have considered all possible partition sizes with the smaller partition at least 30%. To test if this difference of permutations would cause artifacts, we randomly sampled ten hotspots and calculated *P*-values using 'ideal' permutation tests and compared them to the results obtained with the 50/50 permutations. Let us call the two types of *P*-values *P*_1 _and *P*_2_. Of the sampled hotspot-SNP pairs, 756 pairs had *P*_1 _>*P*_2_, and 745 pairs had *P*_1 _≤ *P*_2. _The distribution of differences *P*_1 _- *P*_2 _was very close to the normal distribution centered at 0 (data not shown). Therefore, the test on the sampled hotspots showed that there should be no significant difference between the two types of permutations with regard to *P*-values. However, we reported the more conservative results for an FDR (*q*-value) of 0.01 rather than the customary 0.05, taking into account that the FDR might be slightly underestimated using the 50/50 permutation tests.

### Identifying outlier chromosomes

To deal with the cases when the random Δ*ρ *at a hotspot deviates significantly from the normal distribution, we identified outlier chromosomes using a side score defined as follows. For each permutation, we kept track of which subpopulation the chromosome belongs to. If it is in the subpopulation with the higher hotspot strength, it gains a credit of |Δ*ρ*|; otherwise, it loses by |Δ*ρ*|. The side-score for a chromosome is the sum of the gains and losses, that is:

$$S=\sum_{i\in \Pi }\Delta \rho_{i}$$

where Δ*ρ*_*i *_is positive if the chromosome is in the hotter subpopulation in the *i*th permutation, and negative otherwise. The side-scores of most chromosomes were distributed normally, except for a few outliers. We used Grubbs' method to detect the chromosomes with outlier side-scores, and estimated the *P*-value of being an outlier using the student's *t*-distribution.

### Simulation test

Our simulation program was developed using Python 2.6 and based on simuPOP (version 1.0.3), an open source framework for forward simulation of population genetics [50]. We simulated the evolution of a population of 5,000 individuals for a specified number of generations (for example, 3,000) using a neutral forward-time model. Each individual had a genotype, which consisted of two homologous haplotypes of length 200 kb. Each haplotype was represented by a list of SNPs, each having two alleles 0 and 1. A SNP was generated by a mutation event, simulated using the infinite-site model and Poisson process. When an allele of a SNP became fixed in the population, the SNP was removed from every haplotype.

A hotspot was inserted with its center at 100 kb, and a causal SNP, whose alleles determined different recombination rates at the hotspot, was inserted at various positions (for example, 75, 100 and 125 kb). In each generation, we randomly picked pairs of individuals of opposite sex as parents. For each parent we simulated the meiosis, specifically focusing on the process of recombination, as follows. If both alleles at the causal SNP were cold, then there was no hotspot, and the crossover position was uniformly distributed along the 200 kb. For simplicity, we assumed that there was one crossover in a chromosome of 200 Mb per meiosis; thus, in 200 kb the probability was 0.001. If the causal SNP was heterozygous, then the probability of crossover was increased by ten times the background rate; if both alleles at the causal SNP were hot, then the probability increased by 20 times. Moreover, the crossover position was simulated under normal distribution centered at the position of 100 kb where the hotspot center is located. We also simulated BGC by repairing a small region (called the tract) from the chromosome that initiates a double-strand break by copying from the other chromosome. At the center of the tract was the breakpoint of the crossover, and the tract length was simulated in Gaussian distribution with mean equal to 500. To simulate severe BGC, we increased the mean tract length to 10 kb. After meiosis, the parents transmitted their gamete chromosomes to the next generation.

At the beginning of evolution, the causal SNP had either the cold or hot allele fixed in the population. Then a derived allele was introduced by a mutation event and invaded the population by some evolutionary force. The frequency of the hot allele in the last generation was specified as a simulation parameter. Changes in the hot allele frequency followed a linear trajectory, dictated by the reject-sampling algorithm [51]. If the hot allele frequency decreased during evolution, we call it a 'cooling' model; otherwise, we call it a 'heating' model. At the end of each simulation, a population of genotypes was exported, from which we randomly sampled 10 subsets each of 90 individuals (180 haplotypes) as benchmark SNP data.

### Sliding windows of population split

Some hotspots tend to be near each other, and it is computationally costly to estimate recombination rates. Thus, to avoid redundant splits for closely located hotspots, we searched for association using sliding windows centered at candidate SNPs. Note that we may estimate different recombination rates for the same segment in the overlapping region of two sliding windows, due to the difference of non-overlapping SNPs. We resolved this issue by setting each sliding window to span 500 segments and discarding recombination rates of 50 segments at both ends of the window. As we observed, the estimation of the recombination rate of a segment usually depended on no more than 100 SNPs surrounding it.

### Genomic feature analysis

To search for genomic features associated with candidate SNPs, we first selected candidate and control SNPs as follows. From the output of LDsplit on chromosome 6 of the HapMap JPT + CHB population, we chose split SNPs with *q *≤ 0.01 as candidates and SNPs with *q *> 0.5 as controls. Then, to correct for redundancy of linked SNPs, we clustered SNPs into LD blocks using the ldSelect program [46] with *r*^2 ^≥ 0.8. To control for hotspot-SNP distance, for each hotspot with at least one candidate LD block, we selected control blocks so that the distances between the block centers and the hotspot center were closest to the corresponding distance of the candidate block. For each candidate block, we picked two control blocks, if available, for a more complete coverage of background signals. Then, we selected the SNP from each candidate LD block with the smallest *q*-value as the tag candidate SNP, and similarly from the control blocks. The resulting 498 candidate and 604 control tag SNPs were compared in the following analysis.

First, we extracted 90-base DNA sequences around the candidate and control tag SNPs from human genome reference NCBI b36.2, and uploaded them onto the MEME web server [52,53] to search for conserved motifs, with the minimum number of sites equal to 10 and default values for other parameters. Second, we analyzed genomic features in 200-bp windows extending the candidate SNPs, using the UCSC Table Browser [54] on human genome assembly 'Mar. 2006 (NCBI36/hg18)'. We uploaded the boundaries of windows in the browser extensible data (BED) format as custom tracks, and used the intersection functionality to count the overlapping elements.

## Abbreviations

BGC: biased gene conversion; FDR: false discovery rate; LD: linkage disequilibrium; MAF: minor allele frequency; PPV: positive predictive value; SNP: single nucleotide polymorphism.

## Authors' contributions

JZ, PPK, DRC, and TMP designed the study. JZ implemented all algorithms and performed all computations with the exception of generating genome scale permutation data, which was done by PPK. JZ, PPK, DRC, and TMP analyzed the results. JZ and TMP wrote the paper with input from PPK and DRC.

## Supplementary Material

Additional file 1 **A tab-delimited table in which each row is a hotspot-SNP pair in our original dataset, and columns are positions of hotspots and SNPs, *rs *(reference SNP ID) number of SNPs and LDsplit *P*-values and *q*-values**. Click here for file

Additional file 2 **Source code for the LDsplit program and simulation along with a user's manual**. Click here for file

Additional file 3 **Figures S1 to S4 and Tables S1 to S6**. Click here for file
