# Supplementary material for: Detecting sequence polymorphisms associated with meiotic recombination hotspots in the human genome
Source: Genome Biol. 2010 Oct 20;11(10):R103. doi: 10.1186/gb-2010-11-10-r103 (PMC3218659; doi:10.1186/gb-2010-11-10-r103)
Supplement: Additional file 2 — Source code for the LDsplit program and simulation along with a user's manual. [file gb-2010-11-10-r103-S2.ZIP › LDsplit_manual.pdf]

LDsplit User Manual

July 29, 2010

Introduction

This package contains source code of LDsplit, a novel approach to statistical inference of allele-specific recombination hotspot and a simulation program (see [1] for details). The LDsplit program was written in Perl and runs under Linux, and the simulation script was written in Python and can run under both Windows and Linux.

LDsplit

LDsplit uses the open source program of LDhat version 2.1 [2] as its working horse. The executable files of LDhat are located in the "LDsplit/ldhat_exe/" folder. Because the lk files required for LDhat are very big (about 204 Mb), they are not included in the current source code folder. Users can download the lk files from http://www.stats.ox.ac.uk/~mcvean/LDhat/instructions.html (under "Lookup tables" headline), and copy them to the folder "LDsplit/ldhat_exe/lookup/".

The main script is "LDsplit.pl". It requires two files as input, site and loc files, in the same format as LDhat (examples files are in "test_data" folder). The site file contains SNP alleles in a sample of haplotypes, and the loc file contains the physical positions of segregating sites.

Starting LDsplit.pl under Linux command line shell, user will see a text menu:

```
$ ./LDsplit.pl test_data/test.site test_data/test.loc

Option:
0. Quit
1. Run LDsplit
2. Load LDsplit result data
3. Assess significance of hotspot-SNP association
Your option is:
```

Option 1 is to run LDsplit without computing statistical significance. There are two running modes: one is to run it in parallel under NIH's Biowulf system via swarm command; the other is to run it sequentially on user's computer. The parallel running is tailored for the authors for the sake of speedy computation, and may not be suitable for general users. The sequential running mode can be chosen by all users, but it will take quite long time. The result of LDsplit will be saved in an intermediate data file.

Option 2 loads the result file from option 1 into memory. Then, Option 3 is to assess the statistical significance of hotspot-SNP associations in the window. It allows user to specify boundaries of one hotspot in physical locations, and output p-values of candidate SNPs. Note that Option 3 works only after data have been either generated via Option 1 or loaded from an external file via Option 2.

Simulation

The script for hotspot simulation, "HotspotSimulation.py", was written in Python. It is based on simuPOP version 1.0.3, an open source framework for forward-time simulation of population genetics [3]. User can download simuPOP from its website http://simupop.sourceforge.net/ which contains detailed instructions for installation, user manuals and examples.

This script can be run under either Windows or Linux. Moreover, user can choose to start it in graphic user interface (GUI) or command line. Under GUI mode, user can specify simulation parameters in the dialog box as in Figure 1.

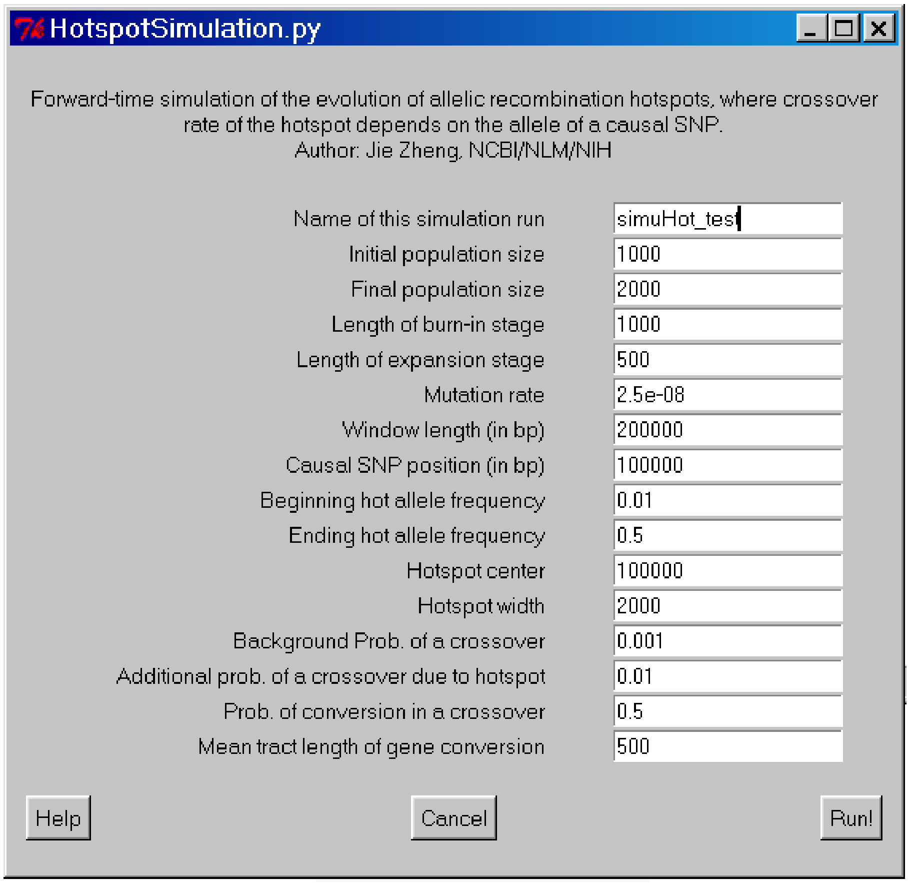

Figure 1. GUI of hotspot simulation script.

With a click on the "Help" button, user will see a detailed description of the options.

Under command line mode, user can use –help option to get description of all options:

```
[zhengj@p3 Simulation]$ python HotspotSimulation.py --help

Forward-time simulation of the evolution of allelic recombination hotspots, where
crossover rate of the hotspot depends on the allele of a causal SNP.
Author: Jie Zheng, NCBI/NLM/NIH
```

```
usage: HotspotSimulation.py [-opt [arg] | --opt [=arg]] ...

options:
  -h, --help
        Show this help message and exit.

  --config=ARG (default: None)
        Load parameters from file ARG.

  --optimized
        Run the script using an optimized simuPOP module.

  --gui=[None|True|False|Tkinter|wxPython] (default: None)
        Which graphical toolkit to use.

  --simuName=ARG  (default: 'simuHot_test')
        A unique string to distinguish the simulation run, for labeling output
        pop file and configure file

  --N0=ARG  (default: 1000)
        Initial population size, to be maintined till end of burn-in stage

  --N1=ARG  (default: 2000)
        Ending population size, after expansion stage

  ...
```

The command line running mode is suitable for batch running and parallel computation.

The output of the simulation is a pop file that contains genotype data of a simulated population. By default, the file name of the pop file is the same as specified to the "simuName" option. User can change it to any other name.

The "Simulation" sub-folder contains another Python script named "PopuSampler.py". It is used for randomly sampling haplotype data from the population file simulated from the HotspotSimulation.py script. User can specify the size of sample (i.e. number of individuals) and the number of samples. Each sample consists of two files, site and loc files, in the same format as input to LDhat and LDsplit. These are the benchmark data that can be used to, say test the performance of LDsplit.

References

1. Zheng, J., et al., *Detecting sequence polymorphisms associated with meiotic recombination hotspots in human genome.* Submitted, 2010.
2. Auton, A. and G. McVean, *Recombination rate estimation in the presence of hotspots.* Genome Res, 2007. **17**(8): p. 1219-27.
3. Peng, B. and M. Kimmel, *simuPOP: a forward-time population genetics simulation environment.* Bioinformatics, 2005. **21**(18): p. 3686-7.
