# Supplementary material for: Detecting sequence polymorphisms associated with meiotic recombination hotspots in the human genome
Source: Genome Biol. 2010 Oct 20;11(10):R103. doi: 10.1186/gb-2010-11-10-r103 (PMC3218659; doi:10.1186/gb-2010-11-10-r103)
Supplement: Additional file 3 — Figures S1 to S4 and Tables S1 to S6. [file gb-2010-11-10-r103-S3.DOC]

**Supplementary Material**

Detecting Sequence Polymorphisms Associated with Meiotic Recombination Hotspots in Human Genome

Jie Zheng^1^, Pavel P. Khil^2^, R. Daniel Camerini-Otero^2 *^ and Teresa M. Przytycka^1*^

*^1^ Computational Biology Branch, NCBI, NLM,* National Institutes of Health *8600 Rockville Pike, Bethesda, MD 20894 Bethesda, Maryland 20894,*

*^2^*Genetics and Biochemistry Branch, NIDDK, National Institutes of Health, 5 Memorial Drive, Bethesda, Maryland 20892, USA*,*

^*^Corresponding authors

R. Daniel Camerini-Otero: email: [camerini@ncifcrf.gov](mailto:camerini@ncifcrf.gov)

Teresa M. Przytycka email: [przytyck@ncbi.nlm.nih.gov](mailto:przytyck@ncbi.nlm.nih.gov)

1. **Distributions of LDsplit *q*-values and *p*-values from real and random splits**

| 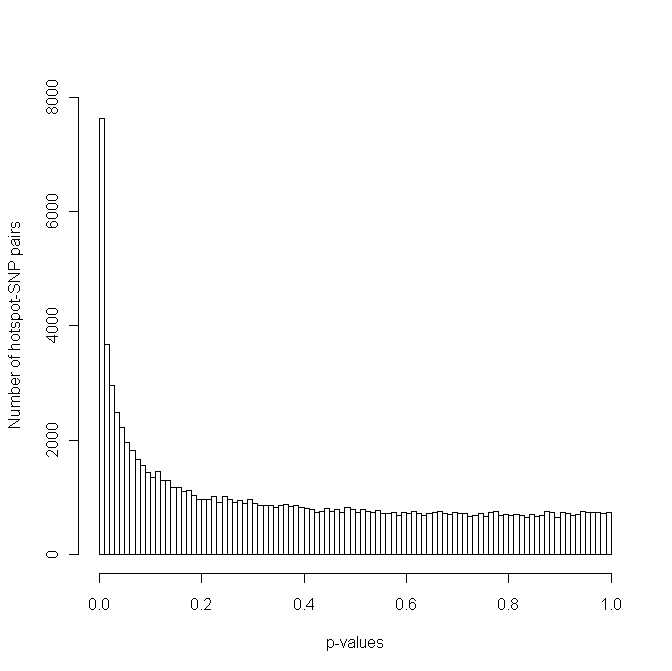   1. Real p-values | 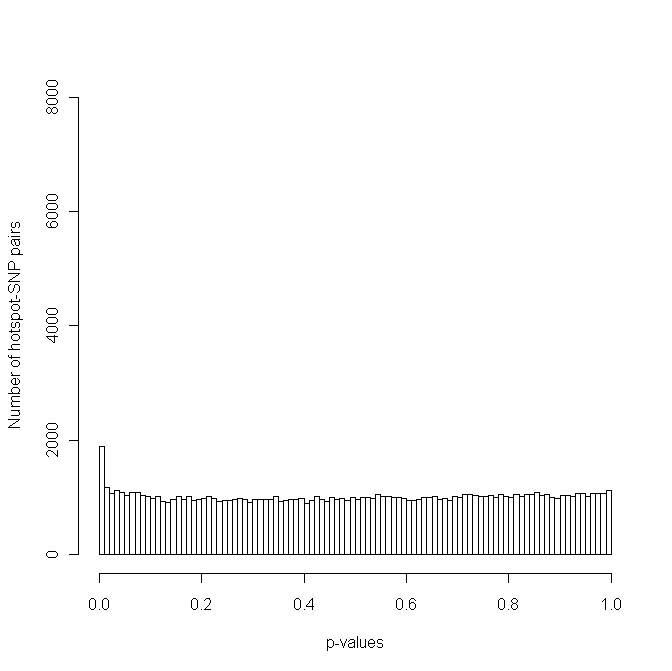   1. Random p-values |
| --- | --- |

**Figure S1.** Distribution of *p*-values of hotspot-SNP pairs in real and random split analyses.

| 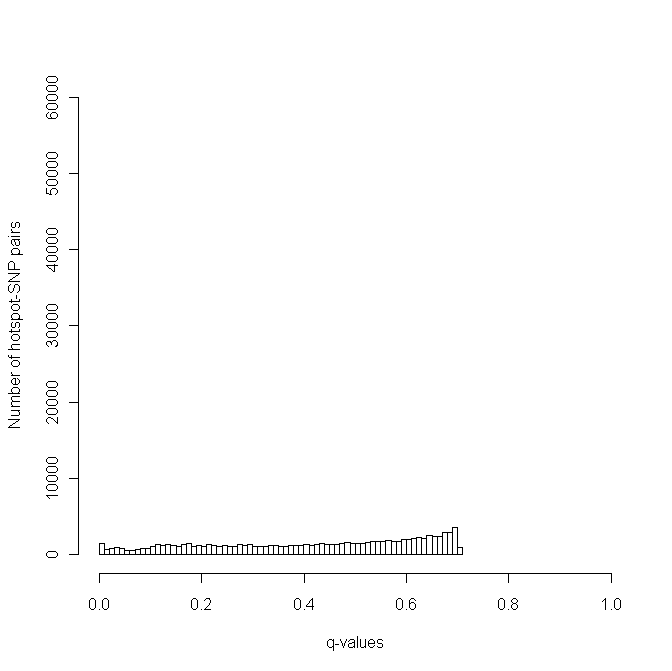  A. Real *q*-values | 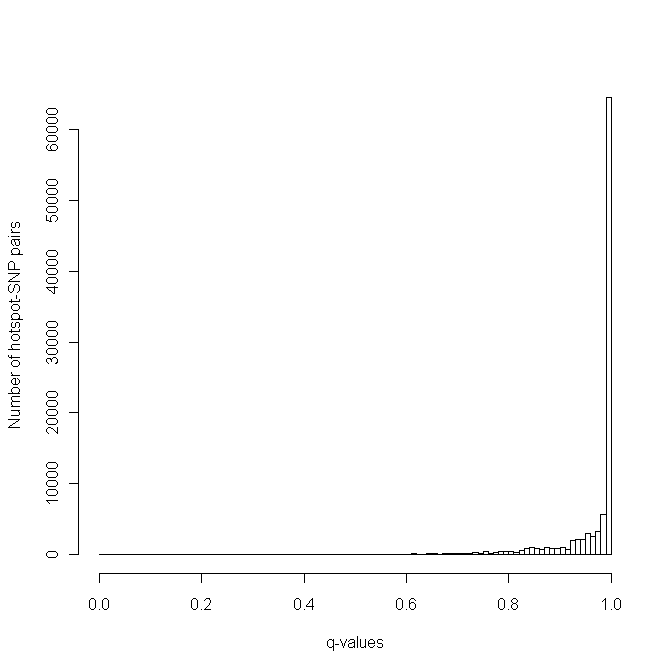  B. Random *q*-values |
| --- | --- |

Figure S2. Histograms of *q*-values of hotspot-SNP associations: (A) *q*-values calculated from splits based on real SNPs. The spike at the left end corresponds to those pairs of significant association (*q* < 0.01); (B) *q*-values from random splits. Compared with the real case, there are almost no hotspot-SNP pairs with small *q*-values and many more with *q*-values close to 1 in the random case.

1. **Distribution of hotspot-SNP distances measured by the number of SNPs**


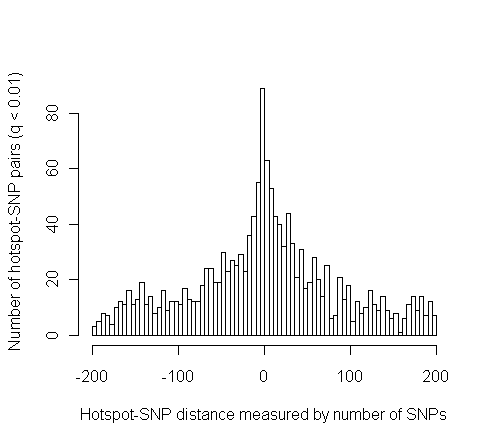


**Figure S3.** Distances between associated SNPs and recombination hotspots of significant pairs (*q* < 0.01) measured by the number of SNPs. When a SNP inside a hotspot, the distance is 0; when a SNP is left side of a hotspot the distance is negative.

1. **Performance of LDsplit on simulated SNP data**

**Table S1**. Performance of LDsplit on simulated data

| **Causal SNP position (kb)** | **Extant hot allele frequency (%)** | **Correct direction prediction (%)** | **# True positive** | **# False positive** | **# True Negative** | **# False negative** | **Specificity (%)** | **Sensitivity (%)** |
| --- | --- | --- | --- | --- | --- | --- | --- | --- |
| **Cooling model** | | | | | | | | |
| **75** | **40** | **60** | **2** | **1** | **6** | **8** | **85.7** | **20** |
| **75** | **45** | **80** | **0** | **6** | **5** | **10** | **45.5** | **0** |
| **75** | **50** | **100** | **8** | **8** | **15** | **2** | **65.2** | **80** |
| **75** | **55** | **100** | **9** | **9** | **7** | **1** | **43.8** | **90** |
| **75** | **60** | **100** | **9** | **11** | **2** | **1** | **15.4** | **90** |
| **100** | **40** | **90** | **8** | **5** | **7** | **2** | **58.3** | **80** |
| **100** | **45** | **90** | **6** | **9** | **20** | **4** | **69.0** | **60** |
| **100** | **50** | **100** | **4** | **7** | **17** | **6** | **70.8** | **40** |
| **100** | **55** | **90** | **6** | **3** | **23** | **4** | **88.5** | **60** |
| **100** | **60** | **100** | **8** | **4** | **12** | **2** | **75.0** | **80** |
| **125** | **40** | **80** | **7** | **13** | **3** | **3** | **18.8** | **70** |
| **125** | **45** | **100** | **8** | **16** | **3** | **2** | **15.8** | **80** |
| **125** | **50** | **100** | **9** | **20** | **10** | **1** | **33.3** | **90** |
| **125** | **55** | **90** | **4** | **22** | **7** | **6** | **24.1** | **40** |
| **125** | **60** | **100** | **8** | **6** | **13** | **2** | **68.4** | **80** |
| **Heating model** | | | | | | | | |
| **75** | **40** | **10** | **3** | **16** | **16** | **7** | **50** | **30** |
| **75** | **45** | **100** | **7** | **10** | **0** | **3** | **0** | **70** |
| **75** | **50** | **100** | **5** | **3** | **24** | **5** | **88.9** | **50** |
| **75** | **55** | **80** | **5** | **11** | **4** | **5** | **26.7** | **50** |
| **75** | **60** | **100** | **10** | **0** | **1** | **0** | **100** | **100** |
| **100** | **40** | **80** | **4** | **15** | **27** | **6** | **64.3** | **40** |
| **100** | **45** | **100** | **10** | **4** | **15** | **0** | **78.9** | **100** |
| **100** | **50** | **80** | **4** | **4** | **8** | **6** | **66.7** | **40** |
| **100** | **55** | **100** | **4** | **14** | **22** | **6** | **61.1** | **40** |
| **100** | **60** | **100** | **10** | **13** | **19** | **0** | **59.4** | **100** |
| **125** | **40** | **78** | **4** | **6** | **15** | **5** | **71.4** | **44.4** |
| **125** | **45** | **90** | **8** | **17** | **18** | **7** | **51.4** | **80** |
| **125** | **50** | **80** | **3** | **4** | **18** | **7** | **81.8** | **30** |
| **125** | **55** | **100** | **9** | **1** | **19** | **1** | **95.0** | **90** |
| **125** | **60** | **100** | **7** | **3** | **22** | **3** | **88.0** | **70** |

**Table S2**. Performance of LDsplit under recurrent mutation

| **Causal SNP position (kb)** | **Extant hot allele frequency (%)** | **Correct direction prediction (%)** | **# True positive** | **# False positive** | **# True Negative** | **# False negative** | **Specificity (%)** | **Sensitivity (%)** |
| --- | --- | --- | --- | --- | --- | --- | --- | --- |
| **Cooling model** | | | | | | | | |
| **75** | **40** | **60** | **3** | **13** | **13** | **7** | **50** | **30** |
| **75** | **45** | **80** | **2** | **14** | **4** | **8** | **22.2** | **20** |
| **75** | **50** | **100** | **7** | **13** | **3** | **3** | **18.8** | **70** |
| **75** | **55** | **90** | **8** | **7** | **5** | **2** | **41.7** | **80** |
| **75** | **60** | **100** | **6** | **12** | **15** | **4** | **55.6** | **60** |
| **100** | **40** | **90** | **7** | **4** | **19** | **3** | **82.6** | **70** |
| **100** | **45** | **100** | **10** | **9** | **20** | **0** | **69** | **100** |
| **100** | **50** | **90** | **5** | **4** | **15** | **5** | **78.9** | **50** |
| **100** | **55** | **100** | **7** | **7** | **10** | **3** | **58.8** | **70** |
| **100** | **60** | **100** | **9** | **11** | **9** | **1** | **45** | **90** |
| **125** | **40** | **90** | **4** | **23** | **4** | **6** | **14.8** | **40** |
| **125** | **45** | **100** | **10** | **0** | **0** | **0** | **NA** | **100** |
| **125** | **50** | **100** | **8** | **21** | **14** | **2** | **40** | **80** |
| **125** | **55** | **100** | **10** | **9** | **9** | **0** | **50** | **100** |
| **125** | **60** | **100** | **10** | **3** | **4** | **0** | **57.1** | **100** |
| **Heating model** | | | | | | | | |
| **75** | **40** | **90** | **7** | **33** | **13** | **3** | **28.3** | **70** |
| **75** | **45** | **80** | **6** | **2** | **22** | **4** | **91.7** | **60** |
| **75** | **50** | **100** | **10** | **17** | **9** | **0** | **34.6** | **100** |
| **75** | **55** | **100** | **10** | **8** | **10** | **0** | **55.6** | **100** |
| **75** | **60** | **90** | **5** | **12** | **8** | **5** | **40** | **50** |
| **100** | **40** | **90** | **7** | **13** | **31** | **3** | **70.5** | **70** |
| **100** | **45** | **90** | **2** | **7** | **13** | **8** | **65** | **20** |
| **100** | **50** | **100** | **7** | **5** | **23** | **3** | **82.1** | **70** |
| **100** | **55** | **100** | **10** | **11** | **21** | **0** | **65.6** | **100** |
| **100** | **60** | **100** | **8** | **10** | **11** | **2** | **52.4** | **80** |
| **125** | **40** | **70** | **0** | **3** | **12** | **10** | **80** | **0** |
| **125** | **45** | **90** | **6** | **15** | **19** | **4** | **55.9** | **60** |
| **125** | **50** | **90** | **8** | **23** | **16** | **2** | **41** | **80** |
| **125** | **55** | **100** | **9** | **5** | **6** | **1** | **54.5** | **90** |
| **125** | **60** | **100** | **9** | **3** | **0** | **1** | **0** | **90** |

**Table S3**. Performance of LDsplit on simulated SNPs with longer biased gene conversion track (10 kb) and with causal SNP position at 100 kb

| **Extant hot allele frequency (%)** | **Correct direction prediction (%)** | **# True positive** | **# False positive** | **# True Negative** | **# False negative** | **Specificity (%)** | **Sensitivity (%)** |
| --- | --- | --- | --- | --- | --- | --- | --- |
| **Cooling model** | | | | | | | |
| **35** | **40** | **1** | **9** | **16** | **9** | **64** | **10** |
| **40** | **100** | **4** | **0** | **0** | **6** | **NA** | **40** |
| **45** | **100** | **6** | **6** | **29** | **4** | **82.9** | **60** |
| **50** | **80** | **4** | **18** | **18** | **6** | **50** | **40** |
| **55** | **100** | **10** | **3** | **23** | **0** | **88.5** | **100** |
| **60** | **100** | **7** | **4** | **9** | **3** | **69.2** | **70** |
| **65** | **100** | **8** | **3** | **6** | **1** | **66.7** | **88.9** |
| **Heating model** | | | | | | | |
| **35** | **60** | **4** | **5** | **8** | **6** | **61.5** | **40** |
| **40** | **40** | **1** | **3** | **34** | **9** | **91.9** | **10** |
| **45** | **90** | **4** | **8** | **38** | **6** | **82.6** | **40** |
| **50** | **100** | **6** | **8** | **19** | **4** | **70.4** | **60** |
| **55** | **100** | **7** | **3** | **25** | **3** | **89.3** | **70** |
| **60** | **100** | **9** | **4** | **15** | **1** | **78.9** | **90** |
| **65** | **70** | **3** | **2** | **14** | **7** | **87.5** | **30** |

1. Genomic feature analysis of candidate SNPs identified by LDsplit

**Table S4**. Enrichment of genomic elements from UCSC genome browser near candidate SNPs

| Element | Candidate | | Control | | p-value (Fisher’s test) | |
| --- | --- | --- | --- | --- | --- | --- |
|  | # hit | # miss | # hit | # miss | 2-tail | greater |
| Self-chain | 178 | 320 | 165 | 439 | 0.00324 | 0.00165 |
| Open chromatin (AoSMC DNase Pk) | 25 | 473 | 17 | 587 | 0.597 | 0.0408 |
| Open chromatin (GM12878 DNase Pk) | 27 | 471 | 22 | 582 | 0.186 | 0.101 |
| OMIM genes | 131 | 367 | 141 | 463 | 0.262 | 0.144 |
| UCSC genes | 29 | 469 | 28 | 576 | 0.413 | 0.226 |
| Broad Histone GM12878 CTCFP | 13 | 485 | 12 | 592 | 0.545 | 0.311 |
| TFBS conserved | 70 | 428 | 78 | 526 | 0.595 | 0.321 |
| Human EST | 41 | 457 | 45 | 559 | 0.653 | 0.355 |
| Switch Gear TSS | 5 | 493 | 4 | 600 | 0.739 | 0.383 |
| Simple repeats | 12 | 486 | 12 | 592 | 0.682 | 0.391 |
| Repeat Masker | 259 | 239 | 319 | 285 | 0.809 | 0.628 |
| STS markers | 6 | 492 | 9 | 595 | 0.797 | 0.746 |
| Evo fold (RNA 2^nd^ structure) | 2 | 496 | 3 | 601 | 1 | 0.747 |
| RepMask 3.2.7. | 268 | 230 | 338 | 266 | 0.503 | 0.78 |
| CpG Islands | 2 | 496 | 4 | 600 | 0.695 | 0.839 |
| Interruped repeats (nested repeats) | 85 | 413 | 116 | 488 | 0.389 | 0.84 |
| Segmental duplication | 6 | 492 | 12 | 592 | 0.348 | 0.897 |
| RGD Human QTL | 445 | 53 | 558 | 46 | 0.0904 | 0.968 |
| Exapted repeats | 0 | 498 | 0 | 604 | NA | NA |
| Microsatellite | 0 | 498 | 0 | 604 | NA | NA |

**Table S5**. Comparing genomic elements overlapping with proximal vs. distant candidate SNPs

| Element | Proximal (< 2 kb) | | Distant (> 50 kb) | | p-value (Fisher’s test) | |
| --- | --- | --- | --- | --- | --- | --- |
|  | # hit | # miss | # hit | # miss | 2-tail | greater |
| Self-chain | 23 | 28 | 32 | 101 | 0.00699 | 0.00512 |
| Open chromatin (AoSMC DNase Pk) | 3 | 48 | 2 | 131 | 0.131 | 0.131 |
| Segmental duplication | 3 | 48 | 2 | 131 | 0.131 | 0.131 |
| Human EST | 11 | 40 | 20 | 113 | 0.378 | 0.199 |
| RGD Human QTL | 46 | 5 | 113 | 20 | 0.473 | 0.251 |
| STS markers | 2 | 49 | 2 | 131 | 0.307 | 0.307 |
| Repeat Masker | 27 | 24 | 65 | 68 | 0.742 | 0.371 |
| RepMask 3.2.7. | 26 | 25 | 67 | 66 | 1 | 0.536 |
| Interruped repeats (nested repeats) | 7 | 44 | 19 | 114 | 1 | 0.622 |
| Switch Gear TSS | 1 | 50 | 2 | 131 | 1 | 0.625 |
| Broad Histone GM12878 CTCFP | 1 | 50 | 2 | 131 | 1 | 0.625 |
| Open chromatin (GM12878 DNase Pk) | 2 | 49 | 6 | 127 | 1 | 0.703 |
| TFBS conserved | 7 | 44 | 22 | 111 | 0.822 | 0.752 |
| OMIM genes | 16 | 35 | 49 | 84 | 0.606 | 0.806 |
| UCSC genes | 2 | 49 | 8 | 125 | 0.729 | 0.819 |
| Simple repeats | 1 | 50 | 6 | 127 | 0.675 | 0.901 |
| CpG Islands | 0 | 51 | 0 | 133 | NA | NA |
| Evo fold (RNA 2^nd^ structure) | 0 | 51 | 1 | 132 | NA | NA |
| Exapted repeats | 0 | 51 | 0 | 133 | NA | NA |
| Microsatellite | 0 | 51 | 0 | 133 | NA | NA |

**Table S6**. Enrichment of repeats from RepeatMasker within 100 bp of candidate SNPs

| Name | Candidate hit | Candidate miss | Control hit | Control miss | p-value (Fisher’s exact test) |
| --- | --- | --- | --- | --- | --- |
| Top 5 repeats enriched near candidate SNP | | | | | |
| MER4D1 | 4 | 494 | 0 | 604 | 0.0414 |
| L1MA7 | 3 | 495 | 0 | 604 | 0.092 |
| MLT1E | 3 | 495 | 0 | 604 | 0.092 |
| L1ME4a | 7 | 491 | 3 | 601 | 0.103 |
| L1MB8 | 4 | 494 | 1 | 603 | 0.182 |
| Top 5 repeats depleted near candidate SNP | | | | | |
| (TG)n | 0 | 498 | 6 | 598 | 0.0268 |
| MIR3 | 0 | 498 | 6 | 598 | 0.0268 |
| L1MB7 | 0 | 498 | 4 | 600 | 0.0898 |
| Charlie8 | 0 | 498 | 3 | 601 | 0.164 |
| HERVH | 0 | 498 | 3 | 601 | 0.164 |
| Top 5 repeats overlapping with highest numbers of candidate windows | | | | | |
| L2 | 32 | 466 | 42 | 562 | 0.809 |
| MIRb | 15 | 483 | 17 | 587 | 0.859 |
| AT_rich | 13 | 485 | 12 | 592 | 0.545 |
| MIR | 10 | 488 | 12 | 592 | 1 |
| AluSx | 9 | 489 | 11 | 593 | 1 |

| 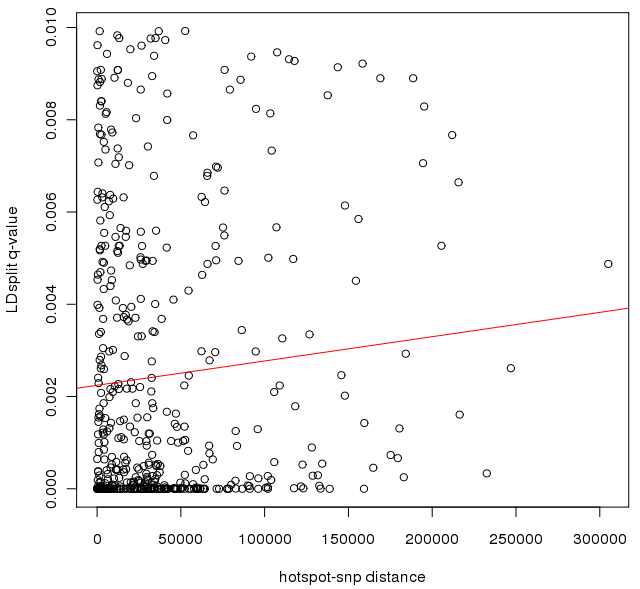  Y = *q*-value,  Pearson’s correlation (greater) 0.0815 (*p* = 0.03463) | 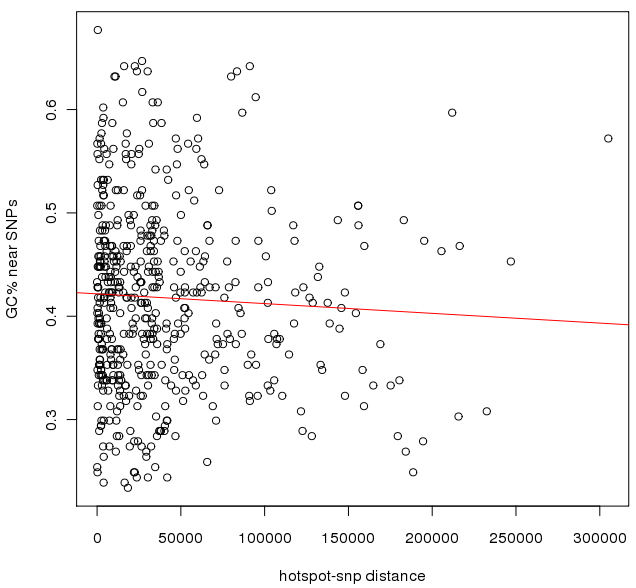  Y = GC% of 200-bp windows  Pearson’s correlation (less)  -0.0503 (*p* = 0.1267) | 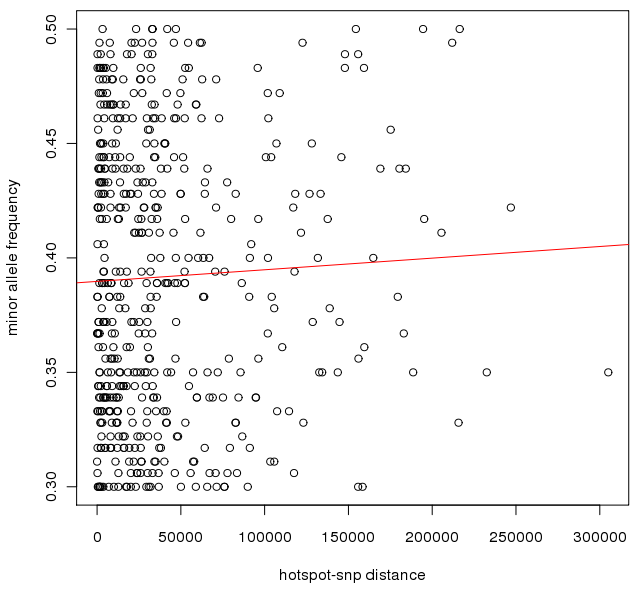  Y = MAF  Pearson’s correlation (greater) 0.0398 (*p* = 0.1828) |
| --- | --- | --- |

**Figure S4**. Correlation of hotspot-SNP distances with LDsplit q-values, GC% around and MAF of candidate tag SNPs.
